# Entropy Analysis of the Thermal Convection of Nanosuspension within a Chamber with a Heat-Conducting Solid Fin

**DOI:** 10.3390/e24040523

**Published:** 2022-04-07

**Authors:** Xuan Hoang Khoa Le, Hakan F. Oztop, Fatih Selimefendigil, Mikhail A. Sheremet

**Affiliations:** 1 Butakov Research Center, National Research Tomsk Polytechnic University, Tomsk 634050, Russia; lexuanhoangkhoa@gmail.com; 2 Department of Mechanical Engineering, Technology Faculty, Fırat University, Elazig 23119, Turkey; hfoztop1@gmail.com; 3 Department of Medical Research, China Medical University Hospital, China Medical University, Taichung 40402, Taiwan; 4 Department of Mechanical Engineering, Celal Bayar University, Manisa 45140, Turkey; fthsel@yahoo.com; 5 Laboratory on Convective Heat and Mass Transfer, Tomsk State University, Tomsk 634045, Russia

**Keywords:** natural convection, entropy generation, nanofluid, solid fin, differentially heated cavity, numerical simulation

## Abstract

Heat transport augmentation in closed chambers can be achieved using nanofluids and extended heat transfer surfaces. This research is devoted to the computational analysis of natural convection energy transport and entropy emission within a closed region, with isothermal vertical borders and a heat-conducting solid fin placed on the hot border. Horizontal walls were assumed to be adiabatic. Control relations written using non-primitive variables with experimentally based correlations for nanofluid properties were computed by the finite difference technique. The impacts of the fin size, fin position, and nanoadditive concentration on energy transfer performance and entropy production were studied. It was found that location of the long fin near the bottom wall allowed for the intensification of convective heat transfer within the chamber. Moreover, this position was characterized by high entropy generation. Therefore, the minimization of the entropy generation can define the optimal location of the heat-conducting fin using the obtained results. An addition of nanoparticles reduced the heat transfer strength and minimized the entropy generation.

## 1. Introduction

Nowadays, the research of many engineers and scientists is related to energy transport enhancement in different technical systems [1,2]. The solution to such a problem can be achieved by using innovative working fluids and extended heat transfer surfaces. Widespread working fluids are mono and hybrid nanofluids [3,4] that can be considered as a suspension of conventional energy transport liquids (water, oil, and others) and solid nanoadditives. Such working fluids can intensify heat transfer processes, and can be considered as an effective replacement of traditional media. Another technique is the addition of extended heat transfer surfaces that include fins, pins, and various solid and porous obstacles [5,6]. The combination of these techniques can be effective for different engineering systems. Thus, Ambreen et al. [7] studied the numerical influence of pin–fin configuration and water-based mono and hybrid nanofluids on the efficacy of a heat sink for an electronic system. Using the ICEM CFD package, the authors investigated heat transport and entropy generation for the considered region under a wide range of governing parameters. It has been shown that hybrid nanofluids can be more effective with optimally shaped fin system. Bahiraei and Monavari [8] investigated nanosuspension energy transfer performance and entropy production in a mini shell and tube thermal exchanger under the influence of nanoparticle geometry and inner fins. The authors obtained the distributions of temperature, velocity and entropy generation rate for different governing parameters. It was revealed that the addition of nanoparticles and fins have an essential influence on heat transfer and entropy generation performance. An experimental and computational analysis of alumina/water nanofluid heat transport performance in a microchannel with a heat sink with fins was conducted by Tiwary et al. [9] for a wide range of Reynolds numbers. Using Ansys Fluent software and an experimental setup, the authors ascertained that boundary layer destruction and secondary vortices appearance is significant for energy transport augmentation. Acharya and Chamkha [10] examined magnetic field influence on nanofluid thermal convection in a hexagonal enclosure with an impact of three inner fins. Using the Galerkin finite element algorithm, the researchers demonstrated that the parallel arrangement of internal fins is more effective, while the heat transport rate can be increased for low values of the Rayleigh number. Manohar et al. [11] scrutinized computationally hybrid nanoliquid circulation on a semi-spherical porous fin. The authors demonstrated that hybrid nanofluids are more effective for heat transfer performance. Other interesting and useful results on nanofluid-forced convection in ribbed channels or channels with finned heat sinks can be found in [12,13,14,15]. In cases of nanofluid behavior in different systems, some results can be found in [16,17,18,19,20].

For nanofluid-free convection in closed chambers with fins mounted on solid walls, some results have been obtained [21,22,23,24,25]. Hatami [21] calculated the thermal convection of water-based nanosuspension in a rectangular chamber with two hot obstacles mounted on the lower border. Using the commercial code FlexPDE, the author revealed a possible rise in the mean *Nu* with alumina nanoparticle concentration. Siavashi et al. [22] computed free convective heat transport and entropy emission of nanosuspension in a square differentially heated enclosure with porous fins located on the vertical hot border. An analysis was conducted using the two-phase nanoliquid model and LTE approach for the porous medium on the basis of the finite volume algorithm. The researchers ascertained that a rise in the Darcy number intensifies the energy transport, while an increase in the fins number has a non-monotonic influence on the strength of energy transport. Al-Farhany et al. [23] examined the computationally MHD free convection of ferrofluid in an inclined porous enclosure, employing the finite element algorithm. The performed study demonstrated that optimal inclination angle and distance between two solid fins augment the energy transport. Hejri and Malekshah [24] conducted a computational study on nanofluid free convection and entropy production in a cooled chamber with inner hot isothermal fins. The influence of *Ra*, fin thickness, and nanoparticle concentration on heat transfer performance was studied. It was ascertained that a growth in fin thickness illustrates a degradation of the fins system. Some interesting results on free convection in enclosures with fins are described in [25,26,27,28,29,30,31].

The performed brief review demonstrates the necessity to analyze the heat transfer performance of engineering systems under the influence of nanofluids and extended heat transfer surfaces in order to intensify energy transport. Moreover, an application of the thermodynamics second law allows us to understand the operability of the system. The objective of this study was to conduct a computational analysis of natural convection and the entropy generation of alumina/water nanofluid in a differentially heated cavity with a heat-conducting solid fin placed on the left hot wall. The effects of fin length and location, as well as nanoparticle concentration on nanosuspension flow, heat transport and entropy production were studied. In this research, a detailed analysis of fin location and length on energy transport performance has been conducted. It should be noted that the novelty of this paper is a detailed analysis of the heat-conducting fin influence on heat transfer performance in the cavity in combination with the thermodynamic second law. The first notion allows us to understand the location and size of the fin for energy transport strengthening, while the second will have a positive influence on the operability of the heat transfer system. It is well-known that entropy generation minimization is an effective technique for the operation improvement of engineering systems.

## 2. Mathematical Model

**Figure 1:**
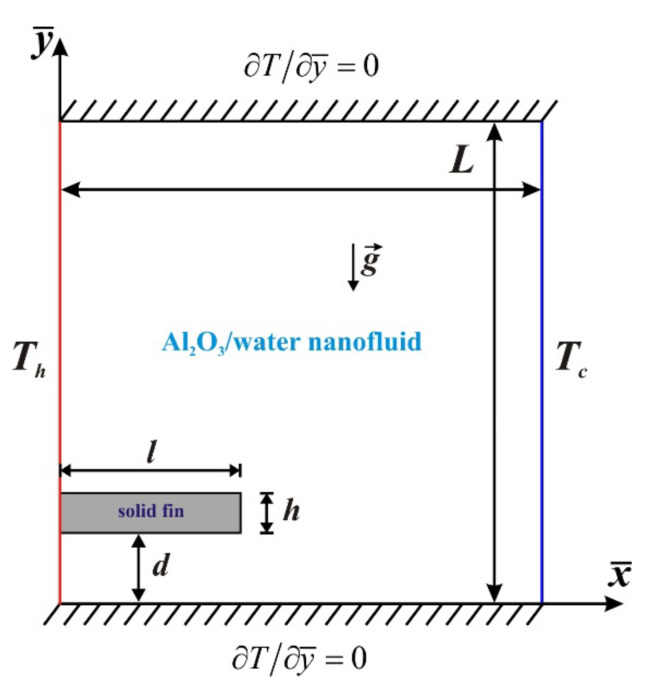
Considered engineering system.

Thermal convection combined with the second law of the thermodynamics of alumina/water nanosuspension in a differentially heated region with a solid fin placed on a hot border is studied computationally. The analyzed domain is demonstrated in Figure 1, where the left wall is isothermal with constant temperature *T_h_* and right wall is isothermal with constant temperature *T_c_* (<*T_h_*). The horizontal walls are adiabatic. The considered nanofluid is a combination of water and tiny solid nanoparticles of alumina. Thermal properties of water and alumina are shown in [32]. Heat equilibrium for the base liquid and nanoadditives is assumed. The Boussinesq model is included in the analysis. The solid heat-conducting fin is placed on the left heated wall. Material of this solid fin is copper.

Control dimensional equations are [32,33]:

- For the nanofluid:

(1) $$\frac{\partial \bar{u}}{\partial \bar{x}}+\frac{\partial \bar{v}}{\partial \bar{y}}=0,$$

(2) $$\rho_{nf}\left(\frac{\partial \bar{u}}{\partial t}+\bar{u}\frac{\partial \bar{u}}{\partial \bar{x}}+\bar{v}\frac{\partial \bar{u}}{\partial \bar{y}}\right)=-\frac{\partial p}{\partial \bar{x}}+\mu_{nf}\left(\frac{\partial^{2}\bar{u}}{\partial {\bar{x}}^{2}}+\frac{\partial^{2}\bar{u}}{\partial {\bar{y}}^{2}}\right),$$

(3) $$\rho_{nf}\left(\frac{\partial \bar{v}}{\partial t}+\bar{u}\frac{\partial \bar{v}}{\partial \bar{x}}+\bar{v}\frac{\partial \bar{v}}{\partial \bar{y}}\right)=-\frac{\partial p}{\partial \bar{y}}+\mu_{nf}\left(\frac{\partial^{2}\bar{v}}{\partial {\bar{x}}^{2}}+\frac{\partial^{2}\bar{v}}{\partial {\bar{y}}^{2}}\right)+g{\left(\rho \beta \right)}_{nf}\left(T-T_{c}\right),$$

(4) $$\frac{\partial T}{\partial t}+\bar{u}\frac{\partial T}{\partial \bar{x}}+\bar{v}\frac{\partial T}{\partial \bar{y}}=\frac{k_{nf}}{{\left(\rho c\right)}_{nf}}\left(\frac{\partial^{2}T}{\partial {\bar{x}}^{2}}+\frac{\partial^{2}T}{\partial {\bar{y}}^{2}}\right);$$

- For the heat-conducting solid obstacle:

(5) $${\left(\rho c\right)}_{s}\frac{\partial T}{\partial t}=k_{s}\left(\frac{\partial^{2}T}{\partial {\bar{x}}^{2}}+\frac{\partial^{2}T}{\partial {\bar{y}}^{2}}\right).$$

Employing stream function $\left(\bar{u}=\frac{\partial \bar{\psi }}{\partial \bar{y}},\bar{v}=-\frac{\partial \bar{\psi }}{\partial \bar{x}}\right)$, vorticity $\left(\bar{\omega }=\frac{\partial \bar{v}}{\partial \bar{x}}-\frac{\partial \bar{u}}{\partial \bar{y}}\right)$, and dimensionless parameters:

(6) $$\begin{array}{c} x=\bar{x}/L,\;y=\bar{y}/L,\;\tau =t\sqrt{g\beta \left(T_{h}-T_{c}\right)/L},\;\theta =\left(T-T_{c}\right)/\left(T_{h}-T_{c}\right),\;u=\bar{u}/\sqrt{g\beta \left(T_{h}-T_{c}\right)L},\\ v=\bar{v}/\sqrt{g\beta \left(T_{h}-T_{c}\right)L},\;\psi =\bar{\psi }/\sqrt{g\beta \left(T_{h}-T_{c}\right)L^{3}},\;\omega =\bar{\omega }\sqrt{L/g\beta \left(T_{h}-T_{c}\right)}, \end{array}$$

the control relations (1)–(5) are rewritten in dimensionless view:

- For the nanofluid-filled chamber:

(7) $$\frac{\partial^{2}\psi }{\partial x^{2}}+\frac{\partial^{2}\psi }{\partial y^{2}}=-\omega ,$$

(8) $$\frac{\partial \omega }{\partial \tau }+u\frac{\partial \omega }{\partial x}+v\frac{\partial \omega }{\partial y}=H_{1}\left(\phi \right)\sqrt{\frac{\mathrm{Pr}}{Ra}}\left(\frac{\partial^{2}\omega }{\partial x^{2}}+\frac{\partial^{2}\omega }{\partial y^{2}}\right)+H_{2}\left(\phi \right)\frac{\partial \theta }{\partial x}$$

(9) $$\frac{\partial \theta }{\partial \tau }+u\frac{\partial \theta }{\partial x}+v\frac{\partial \theta }{\partial y}=\frac{H_{3}\left(\phi \right)}{\sqrt{Ra\cdot \mathrm{Pr}}}\left(\frac{\partial^{2}\theta }{\partial x^{2}}+\frac{\partial^{2}\theta }{\partial y^{2}}\right);$$

- For the heat-conducting solid obstacle:

(10) $$\frac{\partial \theta }{\partial \tau }=\frac{\alpha_{s}/\alpha_{f}}{\sqrt{Ra\cdot \mathrm{Pr}}}\left(\frac{\partial^{2}\theta }{\partial x^{2}}+\frac{\partial^{2}\theta }{\partial y^{2}}\right);$$

The additional restrictions are:

(11) $$\begin{array}{cc} \tau =0: & \psi =0,\;\omega =0,\;\theta =0.5;\\ \tau >0:\\ & \psi =0,\;\frac{\partial \psi }{\partial x}=0,\;\theta =1\;\mathrm{at}\;x=0,\;0\leq y\leq 1,\\ & \psi =0,\;\frac{\partial \psi }{\partial x}=0,\;\theta =0\;\mathrm{at}\;x=1,\;0\leq y\leq 1,\\ & \psi =0,\;\frac{\partial \psi }{\partial y}=0,\;\frac{\partial \theta }{\partial y}=0\;\mathrm{at}\;y=0\;\mathrm{and}\;y=1,\;0\leq x\leq 1,\\ & \left|\begin{array}{l} \psi =0,\\ \frac{\partial \psi }{\partial n}=0, \end{array}\right.\left\{\begin{array}{l} \theta_{nf}=\theta_{s}\\ \frac{\partial \theta_{nf}}{\partial n}=\frac{k_{s}}{k_{nf}}\frac{\partial \theta_{s}}{\partial n} \end{array}\right.\mathrm{at solid obstacle surface} \end{array}$$

These written boundary conditions (11) illustrate that vertical walls are isothermal, while the horizontal walls are thermally insulated. The no-slip boundary condition is assumed at solid surfaces. At the fluid/solid interface (fin surface), the boundary conditions of the forth kind for the temperature (equality of temperatures and heat fluxes) are assumed.

Here, $Ra=\rho_{f}g\beta \left(T_{h}-T_{c}\right)L^{3}/\left(\alpha_{f}\mu \right)$ is the Rayleigh number, $Pr=\mu /\left(\rho_{f}\alpha_{f}\right)$ is the Prandtl number, and $\alpha_{f}=k_{f}/{\left(\rho c\right)}_{f},\;\alpha_{s}=k_{s}/{\left(\rho c\right)}_{s}$ are the heat diffusivities of base liquid and solid fin material. Dimensionless functions of nanoparticles volume fraction [23,24] are $H_{1}\left(\phi \right)=\frac{\mu_{nf}}{\mu_{f}}\frac{\rho_{f}}{\rho_{nf}}=\frac{1+4.93\phi +222.4\phi^{2}}{1-\phi +\phi \rho_{p}/\rho_{f}},\;H_{2}\left(\phi \right)=\frac{{\left(\rho \beta \right)}_{nf}}{\rho_{nf}\beta_{f}}=\frac{1-\phi +\phi {\left(\rho \beta \right)}_{p}/{\left(\rho \beta \right)}_{f}}{1-\phi +\phi \rho_{p}/\rho_{f}}$ and $H_{3}\left(\phi \right)=\frac{k_{nf}}{k_{f}}\frac{{\left(\rho c\right)}_{f}}{{\left(\rho c\right)}_{nf}}=\frac{1+2.944\phi +19.672\phi^{2}}{1-\phi +\phi {\left(\rho c\right)}_{p}/{\left(\rho c\right)}_{f}}$.

It should be noted that viscosity and thermal conductivity of alumina/water nanofluid have been defined using experimental data of Ho et al. [34], as follows:

$$\mu_{nf}=\mu_{f}\left(1+4.93\phi +222.4\phi^{2}\right)\;\mathrm{and}\;k_{nf}=k_{f}\left(1+2.944\phi +19.672\phi^{2}\right)$$

The relation for the mean Nusselt number at the cooled border is:

(12) $$\bar{Nu}=\int_{0}^{1}\left(-\frac{k_{nf}}{k_{f}}\frac{\partial \theta }{\partial x}\right)dy$$

The generation of entropy can be described by employing an influence of viscous dissipation and heat transfer. For the description of dimensional local entropy generation, one can use:

(13) $${\bar{S}}_{gen}=\frac{k_{nf}}{T_{0}^{2}}\left[{\left(\frac{\partial T}{\partial \bar{x}}\right)}^{2}+{\left(\frac{\partial T}{\partial \bar{y}}\right)}^{2}\right]+\frac{\mu_{nf}}{T_{0}}\left[2{\left(\frac{\partial \bar{u}}{\partial \bar{x}}\right)}^{2}+2{\left(\frac{\partial \bar{v}}{\partial \bar{y}}\right)}^{2}+{\left(\frac{\partial \bar{u}}{\partial \bar{y}}+\frac{\partial \bar{v}}{\partial \bar{x}}\right)}^{2}\right]$$

where $T_{0}=0.5\left(T_{c}+T_{h}\right)$.

Relation (13) consists of two parts, namely, the heat transfer part reflecting the entropy production caused by heat transfer $\left({\bar{S}}_{gen,ht}\right)$ and the viscous dissipation part, illustrating the entropy emission caused by the fluid friction $\left({\bar{S}}_{gen,ff}\right)$.

Non-dimensional relation (13) can be defined as:

(14) $$\begin{array}{l} S_{gen}={\bar{S}}_{gen}\frac{T_{0}^{2}L^{2}}{k_{f}{\left(\Delta T\right)}^{2}}=\frac{k_{nf}}{k_{f}}\left[{\left(\frac{\partial \theta }{\partial x}\right)}^{2}+{\left(\frac{\partial \theta }{\partial y}\right)}^{2}\right]+\\ +\chi \frac{\mu_{nf}}{\mu_{f}}\left[4{\left(\frac{\partial^{2}\psi }{\partial x\partial y}\right)}^{2}+{\left(\frac{\partial^{2}\psi }{\partial y^{2}}-\frac{\partial^{2}\psi }{\partial x^{2}}\right)}^{2}\right]=S_{gen,ht}+S_{gen,ff} \end{array}$$

where χ = 10^–3^ [24] is the irreversibility parameter defined as:

(15) $$\chi =\frac{\mu_{f}T_{0}}{k_{f}}\left(\frac{g\beta L}{T_{h}-T_{c}}\right)$$

Dimensionless average entropy emission is:

(16) $$S_{gen,avg}=\frac{1}{\sigma }\int S_{gen}d\sigma =S_{gen,ht,avg}+S_{gen,ff,avg}$$

The local and mean Bejan numbers illustrating the part of heat transport in whole entropy prodution are:

(17) $$Be=\frac{S_{gen,ht}}{S_{gen,ht}+S_{gen,ff}}\;\mathrm{and}\;Be_{avg}=\frac{S_{gen,ht,avg}}{S_{gen,ht,avg}+S_{gen,ff,avg}}$$

## 3. Numerical Method

The formulated Equations (7)–(10) were computed by the finite difference algorithm of second-order accuracy employing the local one-dimensional Samarskii scheme, monotonic Samarskii scheme, and central differences. The employed method has been described in detail in [30,31,32,33]. Moreover, the prepared in-house code has been verified comprehensively employing computational and experimental outcomes of other researchers [30,31,32,33].

**Figure 2:**
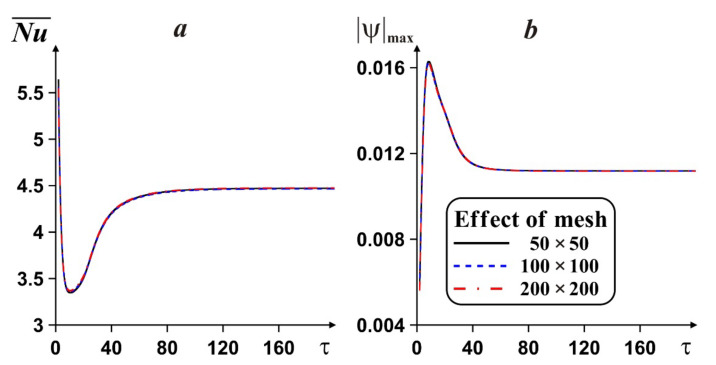
Dependences of the energy transport strength (***a***) and nanosuspension flow strength (***b***) versus the time and grid characteristics.

Mesh sensitivity analysis has been performed for the prepared numerical algorithm in the case of copper fin at ϕ *=* 0.02, *Ra =* 10^5^, *Pr =* 6.82, γ = *l*/*L* = 0.5, δ = *d*/*L* = 0.4, *h*/*L* = 0.1. Figure 2 illustrates an impact of the grid characteristics on mean *Nu* and nanosuspension flow strength.

Observing the weak differences between the analyzed grids for $\bar{Nu}$ and ${\left|\psi \right|}_{\max}$ and the high computational time for the grid of 200 × 200 blocks, the uniform grid of 100 × 100 blocks was selected to calculate the impact of the fin on energy transport performance and entropy emission owing to appropriate accuracy and resolution.

## 4. Results and Discussion

Computations were performed for *Ra =* 10^5^, *Pr =* 6.82, solid copper obstacle, and alumina nanoparticles with ϕ = 0.0–0.04, *h*/*L* = 0.1, δ = 0.1–0.8, γ = 0.1–0.9. This study was devoted to the investigation of energy transport performance and entropy emission considering the impact of nanoparticle concentration, fin length, and position.

**Figure 3:**
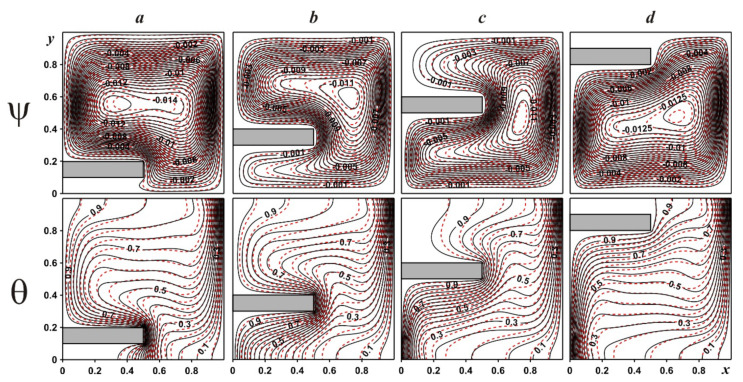
Streamlines and isotherms for γ = 0.5: δ = 0.1—(***a***), δ = 0.3—(***b***), δ = 0.5—(***c***), δ = 0.8—(***d***) (black solid lines are for ϕ = 0.0, red dashed lines are for ϕ = 0.04).

Figure 3 shows streamlines and isotherms within the chamber for γ = 0.5, as well as various locations of the fin and different nanoparticle concentrations. Regardless of the fin position, only one convective cell can be found within the chamber illustrating an appearance of upstream flows close to the left heated border and downstream flows close to the opposite cooled vertical border. An addition of solid fin reflects a deformation of the convective cell, and for δ = 0.3 and δ = 0.5 one can find an appearance of two branches of the flow structure due to a necessity to round this solid obstacle. A variation in the solid fin location characterizes a variation in the convective cell core position. The temperature field allows us to describe the flow structures in detail. It should be noted that for natural convective energy transport, temperature and velocity fields are associated and different features of flow structures can be explained using isotherms patterns. In the present case, heating of the left border and cooling of the right one reflects an appearance of temperature boundary layers along these isothermal borders. An appearance of the temperature stratification zone in the central part characterizes the heating of the chamber from the top zone to the lower part. Taking into account the huge heat conductivity of solid fin material, the location of this solid obstacle illustrates more essential energy removal from the heated wall. The density of thermal boundary layers can be defined using the density of isotherms near the walls. The addition of nanoparticles leads to an increase in the nanosuspension thermal conductivity and dynamic viscosity and, as a result, less intensive heating/cooling can be revealed. The huge density of isotherms close to the lower part of the left border and upper part of the right border is explained by the intensive interaction between the heat flux from the left hot border with the right cooled surface, and the opposite situation can be found for an interaction of the cooled heat flux from the right vertical surface with the hot border.

**Figure 4:**
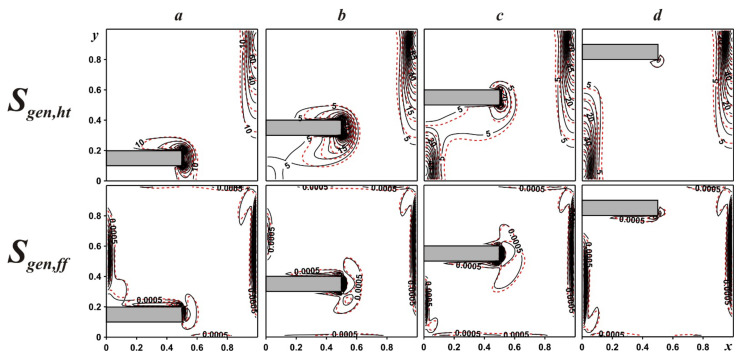
Local entropy emission caused by the heat transport and liquid friction for γ = 0.5: δ = 0.1—(***a***), δ = 0.3—(***b***), δ = 0.5—(***c***), δ = 0.8—(***d***) (black solid lines are for ϕ = 0.0, red dashed lines are for ϕ = 0.04).

An influence of fin location on entropy generation for γ = 0.5 and different fin positions can be found in Figure 4. Areas of entropy emission caused by the heat transport are related to the zones of high isotherm density that can be explained by the high temperature gradient in these zones. Areas of entropy emission caused by the fluid friction are related to velocity boundary layers. These distributions reflect an essential dominance of heat transport irreversibility compared to liquid friction. An introduction of tiny solid particles characterizes small changes, illustrating an increment of diffusion processes.

**Figure 5:**
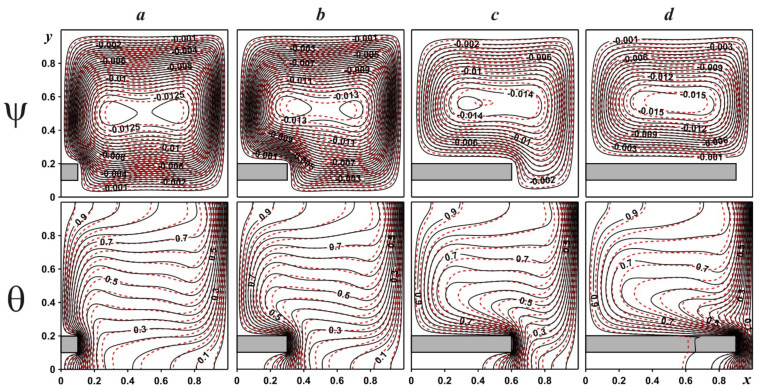
Streamlines and isotherms for δ = 0.1: γ = 0.1—(***a***), γ = 0.3—(***b***), γ = 0.6—(***c***), γ = 0.9—(***d***) (black solid lines are for ϕ = 0.0, red dashed lines are for ϕ = 0.04).

Figure 5 demonstrates isolines of ψ and θ for δ = 0.1 and various lengths of solid fin. The bottom location of the fin is studied due to the high strength of the convective heat transfer for such a position. An appearance of the solid obstacle in the bottom part characterizes a deformation of the convective cell that leads to convective flow intensification and heating from the left vertical wall and solid fin surface. The augmentation of convective heat transport for the bottom location of the fin is related to the heating of the major part of the chamber, and a rise in the temperature drop at the cooled wall. It is interesting to describe the evolution of the thermal boundary layer at the right vertical wall where a thickness of this layer reduces with a rise in fin length. As it has been highlighted above, an addition of alumina nanoparticles reflects an intensification of diffusive processes, namely, a rise in heat conduction strength and viscosity influence.

**Figure 6:**
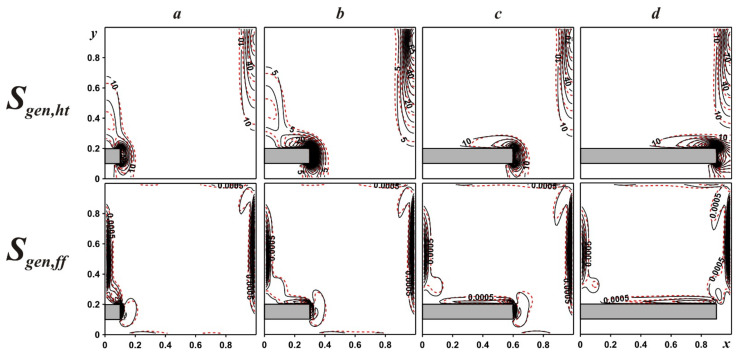
Local entropy emission caused by the heat transport and liquid friction for δ = 0.1: γ = 0.1—(***a***), γ = 0.3—(***b***), γ = 0.6—(***c***), γ = 0.9—(***d***) (black solid lines are for ϕ = 0.0, red dashed lines are for ϕ = 0.04).

The development of entropy generation within the chamber with the growth of the bottom mounted fin is presented in Figure 6. The location of zones with a high density of entropy generation has been described above for Figure 4. It should be noted that for free convective heat transport with low circulation velocity, a contribution of heat transfer irreversibility is a major part for the entropy generation.

**Figure 7:**
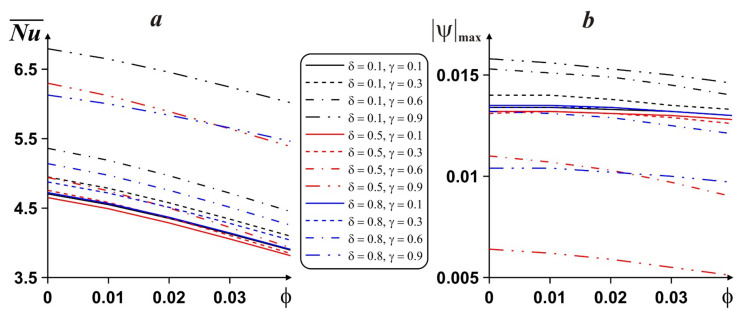
Dependence of average *Nu* (***a***) and liquid motion strength (***b***) on ϕ, δ and γ.

Mean *Nu* behavior and nanosuspension flow strength are shown in Figure 7 for different ϕ, δ and γ. A rise in ϕ for *Ra* = 10^5^ illustrates the degradation of heat transport rate and nanosuspension circulation strength. More essential heat transport intensity and liquid motion rate can be found for a lower position of the fin, and $\bar{Nu}$ rises with γ.

**Figure 8:**
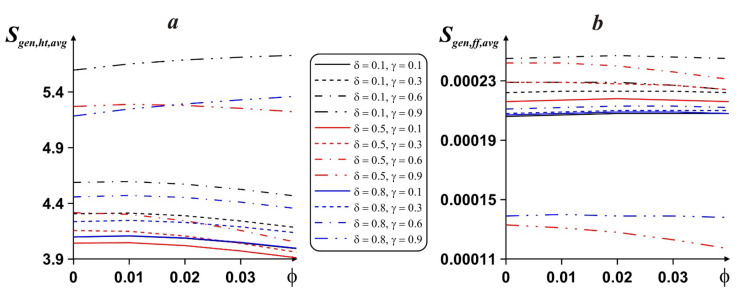
Dependence of mean entropy emission caused by the heat transport (***a***) and liquid friction (***b***) on ϕ, δ and γ.

The nature of entropy emission caused by the heat transport and liquid friction is shown in Figure 8. For the present research, entropy production rate caused by the liquid friction can be neglected when analyzing values of this intensity. At the same time, average entropy emission caused by the energy transport rises with nanoparticle concentration for δ = 0.1, γ = 0.9 and δ = 0.8, γ = 0.9, while for other parameters *S_gen,ht,avg_* reduces with ϕ. *S_gen,ht,avg_* rises with γ, but it is possible to find an optimal location of fin where entropy generation rate can be reduced, e.g., the central location of the fin has such a condition.

About 360 examples of numerical data were generated in this study, encompassing the range of parameters 0.1 ≤ δ ≤ 0.8, 0.1 ≤ γ ≤ 0.9, 0.0 ≤ ϕ ≤ 0.04. These outcomes are employed to define the correlation for the average Nusselt number based on the least-square regression method. As a result, the obtained correlation for the average Nusselt number at vertical wall at steady state for *Ra =* 10^5^, *Pr =* 6.82 and *h*/*L* = 0.1 has the following form:

(18) $$\bar{Nu}=5.441\cdot \delta^{-0.026}\cdot \gamma^{0.099}\cdot {\left(1+\phi \right)}^{-4.854}$$

## 5. Conclusions

The investigation of alumina/water thermogravitational convection combined with entropy generation in a differentially heated enclosure with one solid fin has been conducted computationally, employing the finite difference method. An in-house program code was developed in the present research. The influence of fin length and position, as well as nanoparticle volume fraction, on flow structure, heat transport and entropy emission were studied. Using calculated outcomes, it is possible to highlight that:

- Heat transport strength rises with fin length and has a non-monotonic nature with an increment of the distance between the fin and bottom wall. $\bar{Nu}$ decreases with nanoparticle concentration for *Ra* = 10^5^;
- In engineering systems, a reduction in the entropy generation rate is a major challenge that should be achieved. As a result of the present research, minimum *S_gen,ht,avg_* is defined by the central or upper location of such a fin. The entropy emission caused by this heat transport rises with a growth in fin length;
- An increase in nanoadditive concentration decreases *S_gen,ht,avg_*;
- Correlation for the average Nusselt number in dependence on the fin length and location, as well as nanoparticle volume fraction, has been obtained.

## Data Availability

All data are presented in this article.

## Nomenclatures

*Be*	Bejan number, (–);
*c*	heat capacity, (J·kg^−1^·K^−1^);
*d*	distance from the bottom wall to the fin, (m);
*g*	acceleration due to gravity, (m·s^−2^);
*h*	thickness of the fin, (m);
*H*_1_, *H*_2_, *H*_3_	additional functions, (–);
*k*	thermal conductivity, (W·m^−1^·K^−1^);
*l*	length of the fin, (m);
*L*	size of the chamber, (m);
*Nu*	Nusselt number, (–);
*p*	pressure, (Pa);
*Pr*	Prandtl number, (–);
*Ra*	Rayleigh number, (–);
${\bar{S}}_{gen}$	dimensional local entropy generation, (W·m^−3^·K^−1^);
*S_gen_*	dimensionless local entropy generation, (–);
*S_gen,avg_*	dimensionless average entropy generation, (–);
${\bar{S}}_{gen,ht}$	dimensional local entropy generation due to heat transfer, (W·m^−3^·K^−1^);
*S_gen,ht_*	dimensionless local entropy generation due to heat transfer, (–);
*S_gen,ht,avg_*	dimensionless average entropy generation due to heat transfer, (–);
${\bar{S}}_{gen,ff}$	dimensional local entropy generation due to fluid friction, (W·m^−3^·K^−1^);
*S_gen,ff_*	dimensionless local entropy generation due to fluid friction, (–);
*S_gen,ff,avg_*	dimensionless average entropy generation due to fluid friction, (–);
*t*	time, (s);
*T*	temperature, (K);
*T*_0_	mean temperature, (K);
*T_c_*	cooled wall temperature, (K);
*T_h_*	heated wall temperature, (K);
$\bar{u}$, $\bar{v}$	velocity components, (m·s^−1^);
*u, v*	non-dimensional velocity components, (–);
$\bar{x}$, $\bar{y}$	Cartesian coordinates, (m);
*x, y*	non-dimensional Cartesian coordinates, (–);
**Greek symbols**	
α	thermal diffusivity, (m^2^·s^−1^);
β	thermal expansion parameter, (K^−1^);
γ	non-dimensional fins height, (–);
δ	non-dimensional distance from the bottom wall to the fin, (–);
θ	non-dimensional temperature, (–);
μ	dynamic viscosity, (Pa·s);
ρ	density, (kg·m^−3^);
τ	non-dimensional time, (–);
φ	nanoparticles volume fraction, (–);
χ	irreversibility parameter, (–);
$\bar{\psi }$	stream function, (m^2^·s^−1^);
ψ	non-dimensional stream function, (–);
$\bar{\omega }$	vorticity, (s^−1^);
ω	non-dimensional vorticity, (–);
**Subscripts**	
*c*	cold;
*f*	fluid;
*h*	hot;
*nf*	nanofluid;
*p*	particle;
*s*	solid.
